# Supplementary material for: Improving the Diagnosis of Endometrial Hyperplasia Using Computerized Analysis and Immunohistochemical Biomarkers
Source: Front Reprod Health. 2022 May 12;4:896170. doi: 10.3389/frph.2022.896170 (PMC9580641; doi:10.3389/frph.2022.896170)
Supplement: Supplementary file 1 [file Data_Sheet_1.docx]

**Supplementary Figure 1.** **Proforma used by pathologists for independent assessment of the n=125 tissue samples.**

| **Sample number:**  **Date reviewed: Reviewer:** | | | | |
| --- | --- | --- | --- | --- |
| Adequate endometrial tissue for diagnosis? | **YES** | **NO** | **Only if NO: Tick all that apply** | |
|  | □ | □ | Inadequate Quality | □ |
|  |  |  | Processing / technical problems | □ |
|  |  |  | Other: Specify | |
| **Diagnosis:** | | | | |
| Benign Endometrium | **YES** | **NO** | **Only if YES: only tick one** | |
|  | □ | □ | Atrophic | □ |
|  |  |  | Inactive | □ |
|  |  |  | Proliferative | □ |
|  |  |  | Disordered Proliferative | □ |
|  |  |  | Secretory (including progestin & OCP effect) | □ |
|  |  |  | Menstrual | □ |
|  |  |  | Endometritis | □ |
|  |  |  | Other: Specify | |
| Endometrial Hyperplasia | □ | **WHO 2014** | Hyperplasia without Atypia | □ |
|  |  |  | Endometrioid intraepithelial neoplasia (EIN) | □ |
|  | □ | **WHO 1994** | Simple, non-atypical hyperplasia | □ |
|  |  |  | Complex non-atypical hyperplasia | □ |
|  |  |  | Simple atypical hyperplasia | □ |
|  |  |  | Complex atypical hyperplasia | □ |
| Malignant Neoplasm | □ | □ | Endometrial neoplasm | □ |
|  |  |  | Type: Specify | |
|  |  |  | Other malignant neoplasm | □ |
|  |  |  | Type: Specify | |
| **Polyp:** | | | | |
| Endometrial polyp | **YES** | **NO** | **Only if YES: only tick one** | |
|  | □ | □ | Atrophic | □ |
|  |  |  | Functional | □ |
|  |  |  | Hyperplastic | □ |
| **Notes:** | | | | |

**Supplementary Figure 2** **A representative example of layered segmentation analysis of a section of endometrial tissue containing a region of hyperplasia analysed using the TissueGnostics ‘H&E app’.**

Sections of endometrial tissue biopsises stained with H&E were converted into digital images using a NanoZoomer-XR scanner in 40x mode and stored as NanoZoomer Digital pathology files (.pdpi) which were imported and processed using StrataQuest analysis software. Individual regions of interest (ROI) were identified and analysed using the software to generate a numerical area/volume analysis of different compartments within the tissue. In the image supplied the following colour codes are used: dark green = endometrial stroma; blue = endometrial gland connecting with a lumen; red = endometrial gland without connection to lumen/surface; light green = lumen of a gland.


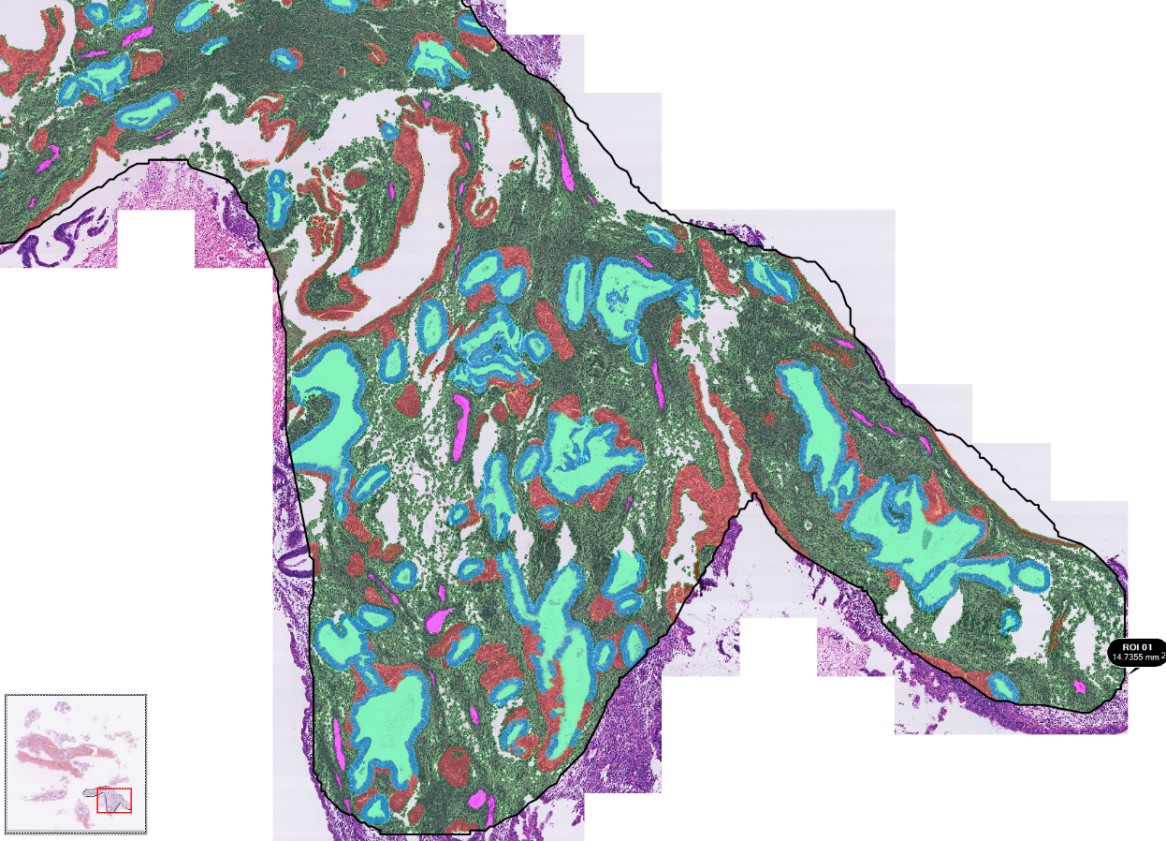


**Supplementary Figure 3. Deficient MMR in a region of tissue diagnosed as EIN**

1. Low power H&E image of an EH lesion containing a focus of EIN (box B), normal endometrium (box C). B) EIN lesion: C) Endometrial glands/normal background tissue. D) Higher power view of the EIN lesion from B showing crowding of glands. B1) Loss of MLH1 expression in the glandular nuclei of the EIN lesion (loss of nuclear brown DAB staining within the glandular nuclei). B2) Normal expression of MSH2 in the same sample. B3) Normal expression of MSH6 in the same sample. B4) Loss of binding partner PMS2 expression in the glandular nuclei of the same sample. D1/ D4) HP images demonstrating loss of MLH1 and PMS2 expression respectively (arrowed), background endometrium positively stained and included in C1/C4 for comparison. D2/D3) HP MSH2 and MSH6 positive expression, background endometrium positively stained and included in C2/C3 for comparison.

G = endometrial gland, S = stroma. Varying magnifications – see scale bars.


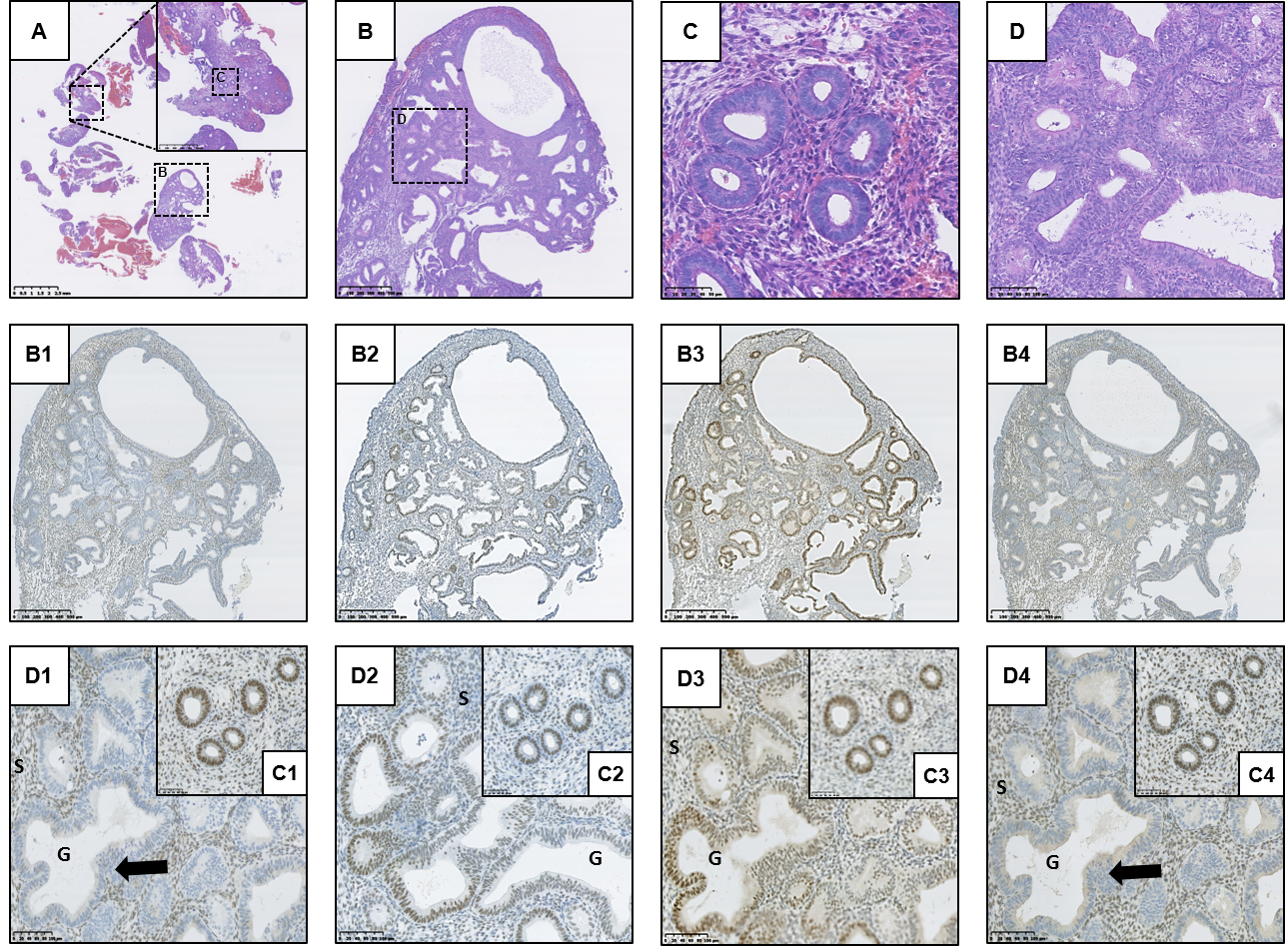


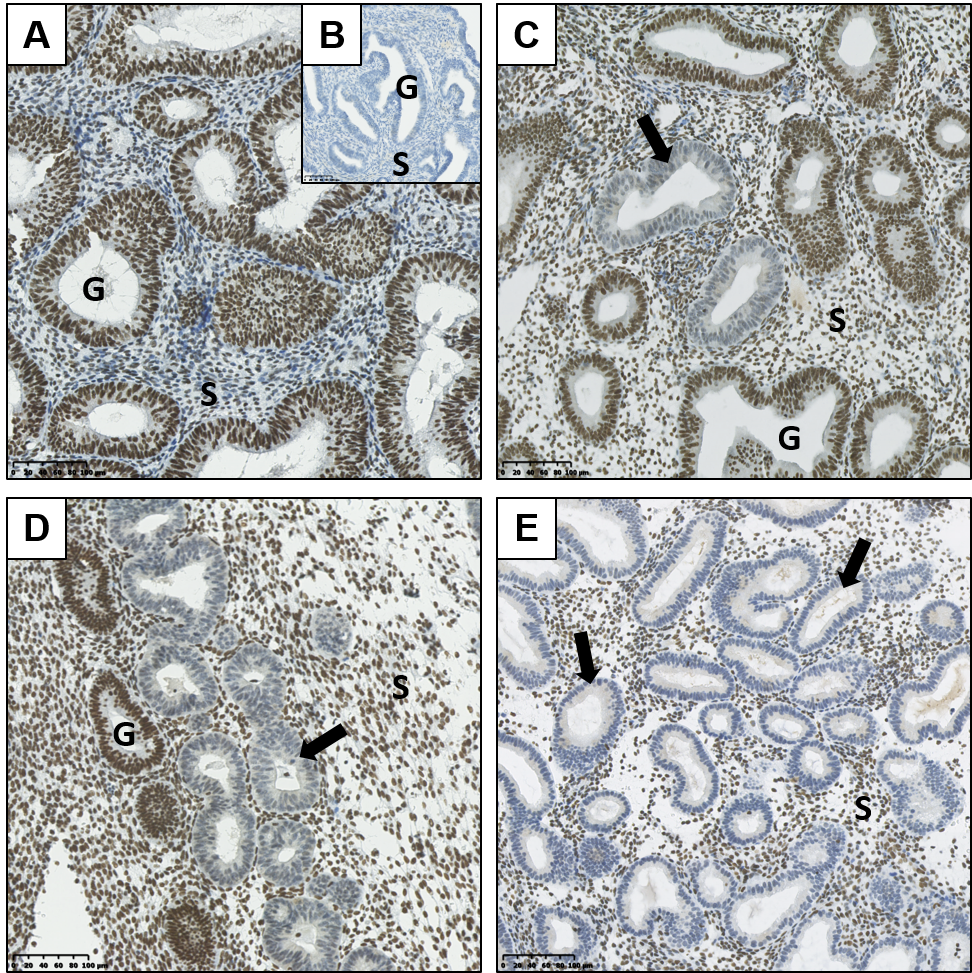


**Supplementary Figure 4.** **Immunohistochemical expression of ARID1A protein within human endometrial hyperplasia (EH) tissue.** A) ARID1A immunohistochemical staining of a tissue section demonstrating hyperplasia without atypia (HwA). Positive ARID1A endometrial glands (G = representative endometrial gland) demonstrated by brown DAB glandular nuclear staining. B) ARID1A negative control. C) Isolated ARID1A null glands (arrow indicating loss of brown DAB glandular nuclear staining) within a tissue section demonstrating Endometrial Intraepithelial Neoplasia (EIN). D) ARID1A confluent null glands (arrow indicating loss of brown DAB glandular nuclear staining) within a tissue section demonstrating Endometrial Intraepithelial Neoplasia (EIN). E) ARID1A complete glandular expression loss (arrow indicating loss of brown DAB glandular nuclear staining) within a tissue section demonstrating Endometrial Intraepithelial Neoplasia (EIN). S = endometrial stromal; used as a positive internal control for ARID1A immunohistochemistry. Varying magnifications – see scale bars.

**Supplementary Figure 5. Immunohistochemical phenotype of Cluster 1 EH cases that progressed to malignancy.**

This analysis focused on the n=3 patients from cluster 1 that had a subsequent confirmed diagnosis of endometrioid endometrial cancer. The image provides a summary of the relevant clinical details, illustrates the appearance of the sample at time of EH diagnosis and the expression patterns of PTEN, PAX2 and HAND2 proteins. The selected images for each case represent serial tissue sections of the most abnormal hyperplastic region.

Key H&E - Haematoxylin and Eosin; PTEN - Phosphatase and Tensin Homolog; PAX2 - Paired Box 2 Protein; HAND2 - Heart and Neural Crest Derivatives-expressed 2.

EC - Endometrial cancer, PMB - Postmenopausal bleeding, HMB - Heavy menstrual bleeding, IMB - Intermenstrual bleeding. Magnifications vary – scale bars are shown.


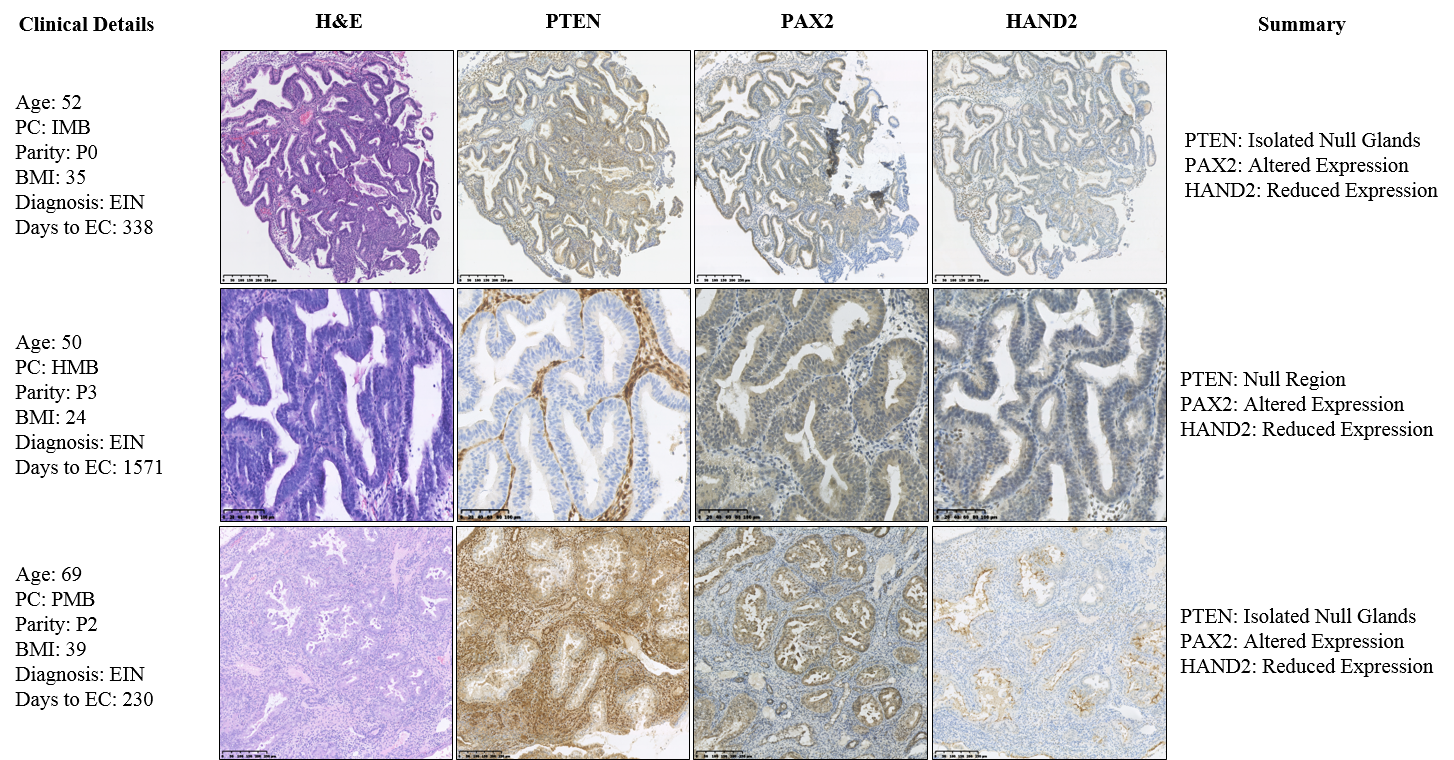


**SUPPLEMENTAL TABLES**

**Supplemental Table 1:** Primary antibodies and detection systems used for chromogenic immunohistochemistry

| **Antigen** | **Species** | **Supplier** | **Cat. No** | **Dilution** | **ImmPRESS™ polymer (DAB)** |
| --- | --- | --- | --- | --- | --- |
| Anti-PTEN (clone 6H2.1)* | Mouse (Monoclonal) | Agilent Dako | M362729-2 | 1:300 | ImmPRESS™ Anti-Mouse (Vector MP 7402) |
| Anti-PAX2 (clone Z-RX2) | Rabbit (Polyclonal) | Invitrogen | 71-6000 | 1:900 | ImmPRESS™ Anti-Rabbit (Vector MP 7401) |
| Anti-ARID1A* | Rabbit (Polyclonal) | Sigma-Aldrich | HPA005456 | 1:2000 | ImmPRESS™ Anti-Rabbit (Vector MP 7401) |
| dHAND (clone M-19) | Goat (Polyclonal) | Santa-Cruz | sc-9409 | 1:250 | ImmPRESS™ Anti-Goat (Vector MP 7405) |
| p53 (clone DO1) | Mouse (Monoclonal) | Santa-Cruz | sc-126 | 1:500 | ImmPRESS™ Anti-Mouse (Vector MP 7402) - DAB |
| MLH1 (clone C-20) | Rabbit (Polyclonal) | Santa-Cruz | sc-582 | 1:100 | ImmPRESS™ Anti-Rabbit (Vector MP 7401) |
| MSH2 (clone FE11) | Mouse (Monoclonal) | Millipore Merck | MABE284 | 1:500 | ImmPRESS™ Anti-Mouse (Vector MP 7402) |
| MSH6 (clone 44) | Mouse (Monoclonal) | BD Biosciences | 610919 | 1:250 | ImmPRESS™ Anti-Mouse (Vector MP 7402) |
| PMS2 (clone A16-4) | Mouse (Monoclonal) | BD Pharmingen | 556417 | 1:300 | ImmPRESS™ Anti-Mouse (Vector MP 7402) |

*Internal positive control present within endometrial tissues.

Supplementary Table 2. Subgroup breakdown recorded within the original index cases in the category of complex atypical hyperplasia (CAH)

| Subgroup within CAH | number | percentage |
| --- | --- | --- |
| Complex atypical hyperplasia | 10 | 41.6 |
| Complex hyperplasia with mild atypia | 6 | 24 |
| Complex hyperplasia with moderate atypia | 4 | 16 |
| Complex hyperplasia with severe atypia | 2 | 8 |
| Severe atypical hyperplasia | 1 | 4 |
| Complex hyperplasia with minor atypia | 1 | 4 |
| Total | 24 |  |

Supplementary Table 3: Demographics and clinical features of patients with a diagnosis of endometrial intraepithelial neoplasia (EIN) or hyperplasia without atypia (HwA) based on a consensus diagnosis by two independent gynaecological pathologists.

|  | EIN  n=52 (%) | HwA  n=54 (%) | P Value |
| --- | --- | --- | --- |
| Age |  |  |  |
| Mean | 52.8 | 52.9 | 0.9898 |
| <40 | 6 (11.6) | 4 (7.4) | 0.7206 |
| 41-50 | 16 (30.8) | 22 (40.8) | 0.3419 |
| 51-60 | 18 (34.6) | 19 (35.2) | 0.7343 |
| 61-70 | 10 (19.2) | 4 (7.4) | 0.9585 |
| >70 | 2 (3.9) | 5 (9.3) | 0.0718 |
| Ethnicity |  |  |  |
| White Scottish | 26 (50.0) | 21 (38.9) | 0.3284 |
| White English | 6 (11.6) | 8 (14.8) | 0.7759 |
| Other | 1 (1.9) | 0 | 0.4906 |
| Not disclosed | 19 (36.5) | 25 (46.3) | 0.3308 |
| Menopausal status |  |  |  |
| Premenopausal | 14 (26.9) | 15 (27.8) | >0.9999 |
| Perimenopausal | 6 (11.5) | 7 (13.0) | >0.9999 |
| Postmenopausal | 30 (57.7) | 30 (55.6) | 0.8428 |
| Unknown | 2 (3.9) | 2 (3.7) |  |
| Presenting complaint |  |  |  |
| PMB | 28 (53.9) | 26 (48.2) | 0.5672 |
| HMB | 13 (25.0) | 16 (29.6) | 0.6657 |
| IMB | 6 (11.5) | 8 (14.8) | 0.7759 |
| Subfertility | 2 (3.9) | 0 | 0.2383 |
| Incidental finding | 3 (5.8) | 4 (7.4) | >0.9999 |
| Parity |  |  |  |
| Nulliparous | 16 (30.8) | 7 (13.0) | *0.0308 |
| 1-4 | 29 (55.8) | 40 (74.1) | *0.0222 |
| >5 | 2 (3.9) | 1 (1.9) | 0.6170 |
| Unknown | 5 (9.6) | 6 (11.1) |  |
| BMI |  |  |  |
| Mean | 37.9 | 38.3 | 0.8661 |
| 21-25 | 5 (9.6) | 2 (3.7) | 0.4425 |
| 26-30 | 2 (3.8) | 5 (9.3) | 0.1964 |
| 31-35 | 8 (15.4) | 2 (3.7) | 0.1411 |
| 36-40 | 9 (17.3) | 8 (14.8) | 0.5687 |
| >40 | 6 (11.5) | 8 (14.8) | 0.2149 |
| Unknown | 22 (42.3) | 29 (53.7) |  |
| Co-morbid factors^ |  |  |  |
| Diabetes mellitus | 12 | 12 | >0.9999 |
| PCOS | 7 | 1 | *0.0264 |
| HRT use | 4 | 7 | 0.5283 |
| Tamoxifen use | 4 | 4 | >0.9999 |
| > 2 of above | 2 | 1 | 0.6060 |

^Complete co-morbid factor information unavailable for n=5 EIN and n=3 HwA patients. Statistical analysis performed using the 2-sided Fisher’s exact test to determine statistical differences between the categorical data and a two-tailed unpaired t-test used to compare the means of the continuous data, for those with EIN and those with HwA. *p<0.05.

**Supplemental Table 4. Summary of ARID1A immunoreactivity and comparison to diagnosis using WHO and EIN criteria**

| ARID1A | Subtype null | EIN | HwA | P value |
| --- | --- | --- | --- | --- |
| N number |  | 51 | 54 |  |
| Positive (%) |  | 45 (88.2) | 54 (100) | 0.011* |
| Null (%) |  | 6 (11.8) | 0 |  |
|  | Isolated glands | 1 (2.0) | 0 | 0.486 |
|  | Confluent glands | 4 (7.8) | 0 | 0.052 |
|  | All glands null | 1 (2.0) | 0 | 0.49 |

^Subgroups of Null ARID1A immunoreactivity included for comparison. HwA = Hyperplasia without Atypia, EIN = Endometrial Intraepithelial Neoplasia. Percentages in brackets. Statistical analysis performed using a Fisher’s exact test, 2-sided. *p<0.05.

#### Supplemental Table 5 Patient characteristics and numbers of endometrial hyperplasia patients broken down by cluster group following immunostaining.

|  | **Cluster 1** | **Cluster 2a** | **Cluster 2b** | **Cluster 3** |
| --- | --- | --- | --- | --- |
|  | N=12 | N=4 | N=54 | N=35 |
| **Age** *(average)* | *51.4* | *51.3* | *53.2* | *52.2* |
| <40 | 3 | 0 | 3 | 4 |
| 41-50 | 2 | 2 | 22 | 12 |
| 51-60 | 5 | 1 | 20 | 11 |
| 61-70 | 1 | 1 | 4 | 8 |
| >70 | 1 | 0 | 5 | 0 |
|  |  |  |  |  |
| **BMI** *(average)* | *35.33* | *34.5* | *38.7* | *39.1* |
| 18.5-25 | 1 | 0 | 2 | 2 |
| 26-30 | 0 | 0 | 5 | 0 |
| >30 | 5 | 2 | 18 | 16 |
| unknown | 6 | 2 | 29 | 17 |
|  |  |  |  |  |
| **Post-**  **menopausal** |  |  |  |  |
| Yes | 5 | 2 | 31 | 21 |
| No | 7 | 2 | 23 | 14 |
|  |  |  |  |  |
| **Presentation** |  |  |  |  |
| PMB | 5 | 2 | 29 | 17 |
| HMB | 4 | 1 | 14 | 10 |
| other | 3 | 1 | 12 | 8 |
|  |  |  |  |  |
| *Nulliparous* | *3* | *2* | *8* | *11* |
| *Parous* | *7* | *1* | *40* | *22* |
| *Unknown* | *2* | *1* | *6* | *2* |

Body max Index (BMI) categorised according to the World Health Organisation (WHO) categories. PMB = Postmenopausal bleeding, HMB = Heavy menstrual bleeding.
